# Editorial: Perinatal Mental Health and Well-Being in Fathers

**DOI:** 10.3389/fpsyg.2022.875620

**Published:** 2022-03-21

**Authors:** Ana Conde, Barbara Figueiredo, Jeannette Milgrom

**Affiliations:** ^1^Portucalense Institute for Human Development (INPP), Portucalense University, Porto, Portugal; ^2^Center for Research in Psychology, School of Psychology, University of Minho, Braga, Portugal; ^3^Parent-Infant Research Institute, Melbourne School of Psychological Sciences, University of Melbourne, Melbourne, VIC, Australia

**Keywords:** fatherhood, anxiety, depression, perinatal period, clinical intervention

Research focusing on fathers' perinatal mental health is recent and less substantial than the research focusing on mothers. Nevertheless, mental health problems and disorders are common in fathers during the perinatal period and should be the focus of attention, namely in planning promotion, preventive, and remedial interventions, with expected benefits for all of the family over time.

The major purpose and potential contribution of this Research Topic is to share conceptual, scientific and practical essays that can inform health care systems and policies about the best practices in the field. The proposals included in this Research Topic reflect the diversity of research aims among studies of fathers' perinatal mental health, namely the development and validation of assessment measures for a range of issues including involvement with the infant (Webb et al.; Pinto et al.), the study of the impact of parental relationship satisfaction on infant development (Nicolaus et al.) and associated processes, such as paternal bonding (Bieleninik et al.; Schaber et al.) and paternal postpartum mental health (Macdonald et al.). The effectiveness and scope of interventions aiming to promote fathers' psychological adjustment and family functioning during parenthood (Rodrigues et al.; Tandon et al.; Battle et al.) and the analysis of the neurobiological correlates of fatherhood (Sobral et al.) are other relevant emerging areas of research.

Consistent evidence supports the impact of fathers' mental disorders on children's mental health and development, on mothers' mental health, and on the overall family functioning. The importance of considering fathers' mental health in perinatal health care and treatments is also consistently suggested. Nevertheless, further research is needed to provide better tools to screen and assess fathers, focusing their specific needs and psychological experience specificities across different cultures. Moreover, a better understanding of the mechanisms and processes involved in father' perinatal psychological (mal)adjustment and parenting should be obtained.

More than being seen exclusively as a source of maternal support, empirical evidence strongly indicates that fathers should be seen as a developing individual, with a particular experience of the phenomena that occur during pregnancy and after childbirth, as well as of the parental role. Using a developmental approach, both the research and clinical practice should promote early (prenatal) identification of fathers at psychosocial risk, exploring the risk and protective factors of their mental health and well-being. Processes associated to father's mental health, as well as processes explaining the impact of father's mental health on children's development and mental health are also essential. Evidence-based interventions addressing the mental health and specific needs of fathers, but also considering all members of the family system, promoting communication, cohesion and mutual support, are still needed. Such promotion, preventive, and remedial interventions are expected to benefit both father's and children's developmental trajectories over time, as well as interrupt the intergenerational transmission of psychosocial risk. At this level, exploring the neurobiological processes involved in parenting is essential and should be prioritized.

In sum, despite the important steps that have been taken in the last two decades in the study and intervention in the perinatal mental health of parents, there is still a long way to go. And at this level, fathers are undoubtedly one of the main actors to consider!

## Author Contributions

All authors listed have made a substantial, direct, and intellectual contribution to the work and approved it for publication.

## Conflict of Interest

The authors declare that the research was conducted in the absence of any commercial or financial relationships that could be construed as a potential conflict of interest.

## Publisher's Note

All claims expressed in this article are solely those of the authors and do not necessarily represent those of their affiliated organizations, or those of the publisher, the editors and the reviewers. Any product that may be evaluated in this article, or claim that may be made by its manufacturer, is not guaranteed or endorsed by the publisher.

